# Cost-effectiveness analysis of alcohol handrub for the prevention of neonatal bloodstream infections: Evidence from HAI-Ghana study

**DOI:** 10.1371/journal.pone.0264905

**Published:** 2022-03-04

**Authors:** Ama Pokuaa Fenny, Evans Otieku, Kwaku Appiah-Korang Labi, Felix Ankomah Asante, Ulrika Enemark

**Affiliations:** 1 Economics Division, Institute of Statistical, Social and Economic Research (ISSER), University of Ghana, Legon, Accra, Ghana; 2 Department of Public Health, Aarhus University, Aarhus, Denmark; 3 Department of Medical Microbiology, University of Ghana Medical School, University of Ghana, Accra, Ghana; London School of Hygiene and Tropical Medicine, UNITED KINGDOM

## Abstract

Published evidence of the cost-effectiveness of alcohol-based handrub (ABH) for the prevention of neonatal bloodstream infections (BSI) is limited in sub-Saharan Africa. Therefore, this study evaluates the cost-effectiveness of a multimodal hand hygiene involving alcohol-based hand rub (ABH) for the prevention of neonatal BSI in a neonatal intensive care unit (NICU) setting in Ghana using data from HAI-Ghana study. Design was a before and after intervention study using economic evaluation model to assess the cost-effectiveness of a multimodal hand hygiene strategy involving alcohol-based hand rub plus soap and water compared to existing practice of using only soap and water. We measured effect and cost by subtracting outcomes without the intervention from outcomes with the intervention. The primary outcome measure is the number of neonatal BSI episode averted with the intervention and the consequent cost savings from patient and provider perspectives. The before and after intervention studies lasted four months each, spanning October 2017 to January 2018 and December 2018 to March 2019, respectively. The analysis shows that the ABH program was effective in reducing patient cost of neonatal BSI by 41.7% and BSI-attributable hospital cost by 48.5%. Further, neonatal BSI-attributable deaths and extra length of hospital stay (LOS) decreased by 73% and 50% respectively. Also, the post-intervention assessment revealed the ABH program contributed to 16% decline in the incidence of neonatal BSI at the NICU. The intervention is a simple and adaptable strategy with cost-saving potential when carefully scaled up across the country. Though the cost of the intervention may be more relative to using just soap and water for hand hygiene, the outcome is a good reason for investment into the intervention to reduce the incidence of neonatal BSI and the associated costs from patient and providers’ perspectives.

## Introduction

The health and economic impact of hospital-acquired neonatal bloodstream infection (BSI) is enormous but often underestimated due to data constraints resulting from a lack of monitoring and reporting from hospital settings [1]. A global estimate of the burden of BSI shows that approximately 3 million neonates suffer the condition annually [1], of which an estimated 25% or more die [2]. In sub-Saharan Africa (SSA) alone, 17 to 29 percent of neonatal mortalities are associated with nosocomial infections. In terms of the economic burden, the annual value of statistical life measured as the disability-adjusted life years (DALY) attributable to neonatal BSI in SSA range between $10 billion and 469 billion [2].

Increasingly, the capacity of neonatal intensive care units (NICU) in under-resourced countries is overstretched due to about 10–15% incidence of hospital-acquired infections (HAI) rate among neonates [3, 4], and the corresponding prolong length of hospital stay (LOS) for treatment of the health condition. The burden of BSI in low and middle-income countries is compounded by limited access to fully-functional laboratories required to enhance early diagnosis and treatment of neonatal sepsis [5].

Consequently, early preventive measures are preferred globally to reduce the associated morbidity and mortality burden but also to reduce the accompanying incremental healthcare cost on families, society, and the health system. Therefore, cost-effectiveness evaluation of interventions aimed at reducing neonatal BSI and to improve quality of life has subsequently been recognized as a major input for health decision making and prioritization [2].

A published guideline for the prevention of hospital-acquired neonatal BSI by the American Academy of Paediatrics highly recommended the use of alcohol-based handrub (ABH) technique for hand hygiene [6]. ABH is a simple hand hygiene technique that requires the use of ethanol-based handrub, especially by healthcare workers [6]. Scientific evidence shows that ABH is microbiologically more effective for the prevention of HAIs than other hand hygiene techniques such as water and soap [7, 8]. For instance, a study finds that compliance with ABH is effective in preventing late onset neonatal BSI [6, 9] and by extension, reduce the associated direct and indirect economic burden it imposes on patients/careers and healthcare providers.

Regardless of the increasing evidence of the potential monetary and health benefits associated with improved hand hygiene care in developed countries, very limited studies in low-and-middle income countries (LMIC) examine the cost-effectiveness of alcohol-based handrub for the prevention of neonatal BSI in NICU settings [10]. In sub-Saharan Africa, and Ghana in particular such evidence is conspicuously limited.

Therefore, this study attempt to measure in monetary terms the cost-effectiveness of a multimodal hand hygiene strategy involving use of ABH compared to existing practice of using only soap and water for the prevention of neonatal BSI in the NICU in a Tertiary Hospital in Ghana. To do so, the study answered a specific question: Is the intervention cost-effective than liquid antiseptic in NICU settings?

## Materials and methods

### Design

We employed a before and after intervention design to conduct a cost-effectiveness analysis (CEA) of ABH based on a simple decision model. For quality check, we report according to the Consolidated Health Economic Evaluation Reporting Standards (CHEERS) checklist for economic evaluation [11]. The study received ethical approval from the Korle-Bu Teaching Hospital. All patients and carers interviewed granted written consent prior to the interviews.

### Setting and location

Study setting was the NICU in the KBTH in Ghana. The hospital serves as the leading tertiary referral hospital in Ghana and it is located at the national capital of Accra. The NICU is managed by the Department of Child Health and has in place an Infection Prevention and Control (IPC) team managed by the Quality Assurance Unit of the hospital. Medical staff at the unit render 24-hour service interspaced by an 8-hour shift system. The unit has three cubicles with each having an admission capacity of 33 neonates at a time. Every year the NICU admits about 0.3% of all newborns in Ghana [12].

### Target population and subgroup

The study population comprises neonates admitted to the neonatal intensive care unit (NICU) of the Korle Bu Teaching Hospital (KBTH). A subgroup of the population (100 in the baseline and 84 in the endline survey) with clinically diagnosed hospital-acquired BSI following positive blood cultures constitute the sample for this study within the two sampling windows (October 2017 to January 2018 and from December 2018 to March 2019). Eligibility for inclusion were BSI neonates with birth weight ≥750 grams and ≤48 hours of age at the time enrolment. Not included in the sample were BSI neonates with severe congenital malformations and those who had undergone surgical procedures. The inclusion and exclusion criteria were contingent on clinical data at the NICU. Sample size calculation was based on 80% power and 0.05 alpha [12].

Two reasons informed the choice of the study population and subgroup. First, previous studies acknowledge the need for more empirical studies to implement cost-effective interventions for neonatal BSI in sub-Saharan Africa [2, 13]. Second, the baseline study shows 28% incidence of neonatal BSI at the hospital compared to previous estimate of about 10% for all healthcare-associated infections in the hospital [4].

### Study perspective

The baseline study (pre-intervention) evaluated the cost of neonatal BSI from patient/carers and provider perspective. Total, average and marginal direct and indirect costs were reported [12]. Consequently, the present study evaluates the cost-effectiveness from both patient and provider perspectives.

### Description of the interventions (comparators)

This study compares the cost-effectiveness of conventional hand hygiene care (CHHC) with optimal hand hygiene care (OHHC) for the prevention and control of neonatal BSI in the NICU. In the NICU of the KBTH, CHHC represents an infection control measure in which healthcare workers practice regular handwashing with liquid antiseptic (soap and water). Caregivers of neonates mostly mothers also make an average of three contacts daily with their newborns for breastfeeding after cleaning their hands with liquid antiseptic, while medical staff (nurses and doctors) establish a countless number of contact with the neonates depending on the severity of their illness. In addition to regular handwashing, medical staff use disposable hand gloves before attending to neonates. Each neonate is kept in a cot/incubator on admission until discharged. Before admission, cots and incubators are disinfected by trained health workers to prevent bacterial transmission. All the eligible 100 participants recruited during the baseline survey received conventional hand hygiene care. Compliance was generally followed as reported elsewhere [14]. The baseline survey lasted four months, spanning October 2017 to January 2018. For the four months of data collection, an amount of US$ 3,236 was spent on liquid antiseptic by the NICU, equivalent to 21.1% of the total cost of the CHHC intervention for the same period. The unit cost of the CHHC intervention estimated from the overall sample of 357 and the observed average LOS of 13 days is $3.30 per day.

### The intervention

At the end of the baseline survey, a multimodal hand hygiene intervention was initiated by the NICU. It involves CHHC plus ABH, referred to in this paper as optimal hand hygiene care (OHHC). At the NICU, ABH equals 70% formulation of alcohol-based handrub used to prevent bacterial transmission and bloodstream infection among neonates. The choice of intervention followed the World Health Organisation’s multimodal hand hygiene strategy for the prevention of infectious diseases including neonatal BSI [15]. The implementation of the OHHC was systematic. First, the result from the baseline study was used to create awareness among medical staff about the 28% incidence and the associated cost of neonatal BSI in the NICU. Second, a 70% alcohol formulation was prepared by the hospital pharmacy and made available for use by the medical staff (doctors and nurses) and caregivers of neonates at the point of care. Additionally, soap and water were made available to enhance multimodal hand hygiene. Medical staff also used disposal medical hand gloves before attending to neonates. Compliance with recommended checklist [14] for hand hygiene was periodically monitored and evaluated to ensure due diligence was followed. Eleven months into the OHHC intervention, the endline survey was undertaken to evaluate the cost-effectiveness of both the baseline and endline interventions. All the eligible 84 participants recruited during the endline survey received optimal hand hygiene care. Data on direct and indirect cost of neonatal BSI was collected for another four months (December 2018 to March 2019).

### Decision model

**Fig 1:**
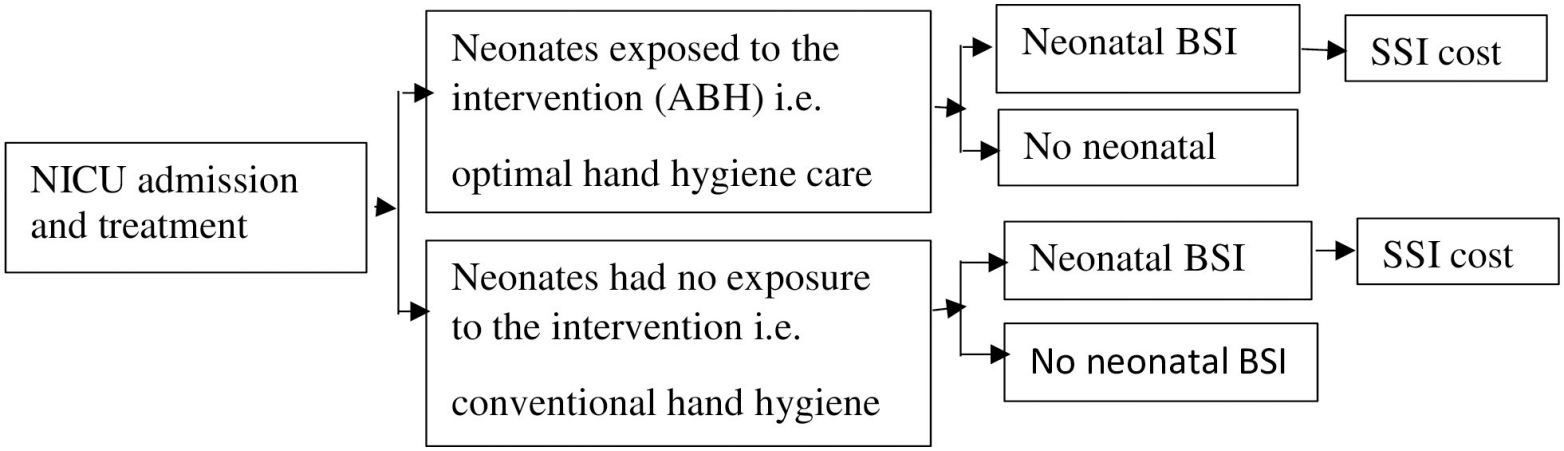
Decision tree.

We used a simplified decision tree model comparing a cohort of neonates with and without exposure to the intervention (Fig 1). Our model assumption is that if the optimal hand hygiene care is successful, there will be a reduction in the risk of neonatal BSI and the consequential costs. For instance, available studies on the efficacy of ABH suggests it is more effective in preventing transient pathogens from healthcare workers’ hand to patients such as neonates than other hand hygiene strategies, including soap and water [16, 17]. We derived our model parameters from the observed probability of neonatal BSI and the estimated costs before and after the intervention plus the intervention cost (Table 1). We applied a 2.5% discount in comparing the estimated cost before and after the intervention. Thus, our time horizon and discounting was limited to the period of the study and did not consider future costs.

**Table 1:** Model parameters.

Parameters	Value [95% CI]	Source
Probability of neonatal BSI before intervention (%)	28 [23–33]	Fenny *et al*. [12]
Probability of neonatal BSI after intervention (%)	18 [14–21]	Patient data*
Patient cost of neonatal BSI before intervention (USD)**	1,026 [902–1,150]	Fenny *et al*. [12]
Patient cost of neonatal BSI after intervention (USD)	599 [563–642]	Patient data*
Hospital cost before intervention (USD)	1,010 [758–1,309]	Fenny *et al*. [12]
Hospital cost after the intervention (USD)	520 [497–588]	Patient data*

*Estimate from patient data after the intervention study

**USD—United States Dollars.

### Measurement of effectiveness

The primary effect measure is the proportion of neonatal BSI cases avoided with the ABH (the risk of neonatal BSI averted) and the attributable costs. We argued that the number of mortalities avoided and life years lost based on the estimated change in mortality risk is more uncertain due to the limited number of observations. Therefore, measurement of effectiveness assumed the incremental outcomes related to the intervention and calculated as:

$$\text{Effectiveness}=\left[After-intervention\;BSI_{outcome}-Before-intervention\;BSI_{outcome}\right]$$ (1)

### Estimating resources and costs

To analyse the attributable cost of neonatal BSI, we matched BSI neonates with non-BSI neonates based on birth weight, sex, and delivery type [12]. For the purpose of precision and generalisability, activity-based micro-costing approach (ingredient costing method) was employed to capture and measure both patient and hospital cost [12]. Direct patient cost data covered medical and non-medical costs incurred by neonates and their caregivers for the period neonates were on admission and the subsequent 30 days after discharge. The direct medical cost includes the cost of laboratory tests, medical consultation, review cost, and the cost of drugs. The non-medical direct cost includes the cost of transportation and feeding.

For indirect cost, the study measured the opportunity cost of productive lost days to parents/caregivers of neonates related to hospital admission i.e. the loss of income during admission and the subsequent 30 days after discharge. For BSI neonates who died before the 30- day post-discharge surveillance, cost was captured up to the time of death. Further detail is captured in the baseline study [12].

We estimated hospital cost per BSI neonate as the product of the daily hospital cost and the extra LOS of 10.2 and 5.1 days as derived before and after the intervention, respectively. The procedure used for calculating the hospital cost is reported in the baseline paper [12]. Briefly, the method involves an activity-based gross costing that captures the sum of all recurrent and annualized capital expenditures incurred by the NICU within the 2017/2018 and 2018/2019 financial years when the pre and post-intervention studies took place. The recurrent cost includes staff remuneration, cost of clinical support, and all other consumable items used by the NICU. The capital cost comprises the annualized expenditures of office space, baby cots/incubators, etc.

The cost of the intervention was calculated as the sum of all expenses incurred during the intervention, and which will be necessary for continuation of the intervention. It includes the cost of alcohol hand rub and supplies, 200 litre of ethanol, staff training allowance, etc. Cost of materials were valued at market price and the staff training allowance valued using a per diem amount.

The data was collected prospectively by a team of experts in health economics with funding from the Danish Ministry of Foreign Affairs. Informed consent was sought from all participants. The study was granted ethical approval by the Institutional Review Board of the Korle Bu Teaching Hospital. Therefore, the robustness and reliability of the data lies in its credibility and quality.

### Statistical analysis

First, statistical background differences in characteristics of the participants were measured using chi-square statistics for categorical variables (p<0.05). This is to ensure that participants in each group were comparable. Second, we computed our primary effect based on z-test of proportions and estimated the 95% confidence intervals (95%CI) due to the slightly differences in sample size between groups. To preserve our crude estimate based on the limited sample size, we decided to report the 95% CI from the z-test. We also analysed and reported the mean and 95% CI for three indicators of severity i.e. LOS, number of blood culture tests, and outpatient visits.

The incremental cost savings with the intervention was computed as the difference in patient total cohort cost i.e. post-intervention patient cost minus pre-intervention patient cost. To understand the potential impact of the ABH intervention, the estimated incremental cost was multiplied by an estimated annual number of neonatal BSI in Ghana. To examine the incremental hospital cost attributable to neonatal BSI, the estimated daily hospital cost of $99 and $102 from the pre and post intervention per neonate was multiplied by the mean extra LOS attributable to neonatal BSI. Again, we populated our decision model with the estimated parameter values using after intervention cohort as basis for calculating cost and effects with and without exposure to the intervention.

An incremental patient cost-effectiveness ratio (ICER) was analysed by diving the change in patient total cohort cost obtained from the pre and post-intervention by the number of neonatal BSI cases avoided i.e. the effect gain [18–20]. The calculation of ICER assumed the formula:

$$ICER=\left(C_{1}-C_{0}\right)/\left(E_{1}-E_{0}\right)$$ (2)

Cost calculations were made in Ghana cedis (GH₵) and converted to 2019 purchasing power parity in United States Dollars (US$) using a web-based purchasing power parity convertor that equates the PPP value of US$1.00 to GHC1.645 [21]. The data was processed and analysed in both STATA version 14.0 and Microsoft Excel.

### Sensitivity analysis

The robustness of the incremental cost derived from the intervention was analysed for uncertainty using both one-way and multiway deterministic sensitivity analyses (DSA). We limited the sensitivity analysis to DSA because the 95% uncertainty intervals for our effect estimate did not overlap, which makes it enough to conclude on the effectiveness of the intervention. The input parameters include patient cost, hospital cost, and the probability of neonatal BSI pre and post-intervention. The parameters were varied using the minimum and maximum values of the 95% uncertainty intervals around the base case mean cost. Knowing how the uncertainty intervals derived from the 95%CI affect the incremental cost-effectiveness of healthcare interventions is highly recommended in the WHO guide to cost-effectiveness analysis [22]. We used Microsoft Excel to build a sensitivity analysis table based on our decision tree model.

## Results

A total of 357 and 469 neonates were admitted to the NICU during the pre and post-intervention surveys. The incidence of neonatal BSI was 28% [95% CI: 23%–33%%] and 17.9% [95%CI: 14%–21%] in the pre and post intervention groups respectively. More than half the sample were male neonates, and more than 80% and 60% were born through caesarian deliveries in the pre and post-interventions respectively. The majority of neonates were born low birth weight (≥1kg–≤2kg). There were statistically no significant differences in the background characteristics of the sub-sample for both periods (Table 2).

**Table 2:** Background characteristics of neonates.

	Pre-intervention		Post-intervention		
	Total (n = 357)	BSI neonate (n = 100)	Total (n = 469)	BSI neonate (n = 84)	P value
Risk of neonatal BSI [95%CI]		28% [23%–33%]		17.9% [14%–21%]	
Sex of neonates					0.922
Male	202 (56.6)	61 (61.0)	247(52.7)	43(51.2)	
Female	155 (43.4)	39 (39.0)	222(47.3)	41(48.8)	
Mode of delivery					0.371
Spontaneous vaginal delivery	49 (13.7)	12 (12.0)	172(36.7)	32(38.1)	
Caesarian delivery	308 (86.3)	88 (88.0)	297(63.3)	52(61.9)	
Birth weight					0.116
Extremely low birth weight (<1kg)	15 (4.2)	2 (2.0)	27(5.8)	9 (10.7)	
Low birth weight (≥1kg-≤2.5kg)	212 (59.4)	51 (51.0)	255(54.4)	54 (64.3)	
Normal (≥2.6kg-≤4.0kg)	112 (31.4)	42 (42.0)	162(34.5)	20(23.8)	
Macrosomia (>4kg)	18 (5.0)	5 (5.0)	25(5.3)	1(1.2)	

Kg—kilogram; 95% CI—95% confidence interval.

### Incremental outcomes

For all the incremental outcomes analysed, there was a reduction in the number of events following the implementation of the ABH intervention. Example, the rate of neonatal BSI was 28% in the baseline as opposed to 17.9% in the endline study, resulting into 16% avoidable neonatal BSI episodes. Likewise, the study recorded 73.1% reduction in BSI-attributable mortalities, 50% reduction in the mean additional LOS for BSI neonates, 43.8% reduction in the mean number of outpatient visits, and 52.1% reduction in the mean number of blood cultures for BSI neonates (Table 3).

**Table 3:** Incremental outcomes between CHHC and OHHC.

Sample description and outcome	Pre-intervention (CHHC)	Post-intervention (OHHC)	Incremental (OHHC-CHHC)
Total mortalities recorded in the overall sample	71 (19.9)	89 (19.0)	-
Total number of neonatal BSI cases identified	100 (28.0)	84 (17.9)	-16 (16.0)
Total mortalities among BSI neonates	26 (26.0)	7 (8.3)	-19 (73.1)
Mean LOS for the overall sample	13 [11.6–14.4]	11.4 [10.3–12.4]	-1.6 (12.3)
Mean additional LOS for BSI neonates	10.2 [9.9–10.5]	5.1 [4.8–5.4]	-5.1 (50.0)
Mean number of outpatient visits for BSI neonates	3.2 [2.6–3.5]	1.8 [1.3–2.2]	-1.4 (43.8)
Mean number of blood cultures for BSI neonates	2.6 [2.3–2.8]	1.3 [1.1–1.6]	-1.36 (52.1)

**Note**: [95% CI]; (%).

### Intervention cost

The total cost of the intervention was covered with funds for the HAI-Ghana project. Approximately 66% of the intervention cost was staff-related expenses, while the remaining 34% went into the purchase of standardized supplies (60 concentrates, 8 wall mounts, 100 dispensers, 200 litres of ethanol, etc.). The per neonate cost of the intervention per day estimated from the overall sample of 469 and the observed average LOS of 11.4 days is $3.60 per day (Table 4).

**Table 4:** Distribution of hand hygiene care cost at the NICU in US$ (2019 PPP-adjusted).

Description	Quantity	Unit price	Total (CHHC)	Total (OHHC)
**Fixed Cost**				
Notice board for feedback	1	44	44	44
Hand hygiene posters	3	12	36	36
Medical hand gloves (100pcs/box)	17 boxes	42.41	721	721
Hand washing sink	1	100	100	100
Hand towel	100	2.32	232	232
*Total*			1,133	1,133
**Variable Cost**				
Alcohol hand rub and supplies*	-	-	-	5,396
200 litres of ethanol	1L	3.75	-	750
Fixing of wall mounts	8	1.37	-	11
Staff training allowance	10	71.60	-	716
BSI surveillance/supervision (2 staff)	8 months	-	10,848	11,203
Liquid antiseptic (soap & water)	-	-	3,192	-
Disposable tissue for hand wipe	200packs	0.96	192	-
*Total*			14,232	18,076
***Grand Total***			15,329	19,209
***Cost per day for each neonate***			3.30	3.60

*Includes 60 concentrates, 8 wall mounts and 100 dispensers.

#### Cost-effectiveness analysis

We disaggregated the patient cost of healthcare at the NICU into medical and non-medical costs. The medical cost relates to antibiotics, review/outpatient care, laboratory tests/blood culture, and medical consultation. Non-medical cost includes transportation, accommodation and feeding. In all the categories of cost analysed, there was a reduction following the implementation of the OHHC. For instance, the mean total cohort cost of hospitalization per BSI neonate under CHHC scenario was US$1026 as opposed to US$598.63 in the OHHC scenario. The difference resulted in $427.4 patient cost savings, equivalent to 41.7% cost saving per BSI neonate (Table 5). Applying the 17.9% incidence of neonatal BSI observed during the intervention to the total NICU admissions of 2,142 in 2019 will yield approximately 383 neonatal BSI cases annually. Therefore, multiplying $427.4 by the estimated annual neonatal BSI cases of 383 will yield $163,694.2 patient cost saving annually if the intervention is sustained at the NICU. The incremental hospital cost attributable to BSI was $1,010 for CHHC and $520 for OHHC, resulting in a cost saving of $490 per neonatal BSI episode with the intervention, and consequently, an annual hospital cost savings of $187,670.

**Table 5:** Attributable cost of neonatal BSI (2019 PPP-adjusted USD).

	Pre-intervention [95%CI]	Post-intervention [95%CI]	Difference (% change)
Patient direct medical costs			
Mean cost of systemic antibiotics	135.87 [119–153]	73.36 [68–79]	-62.51 (46.0)
Mean cost of review (Out-patient visits cost)	74.89 [52–97]	35.26 [28–42]	-39.63 (52.9)
Mean cost of laboratory tests/Blood cultures	127.4 [118–137]	82.38 [79–86]	-45.02 (35.3)
Mean cost of consultation*	207.84 [206–209]	207.84 [206–209]	0
Patient indirect medical cost			
Mean non-medical cost**	203 [159–248]	87.46 [80–95]	-115.54 (56.9)
Mean cost of productivity loss	277 [248–306]	112.33 [102–122]	-164.67 (59.4)
Mean patient cost per BSI neonate	1,026 [902–1,150]	598.63 [563–642]	-427.37 (41.7%)
Hospital cost attributable to neonatal BSI	1,010 [758–1,309]	520 [497–588]	-490 (48.5%)

*Fixed cost

**Includes cost of transportation, feeding & accommodation for mothers of neonates with BSI.

#### Cost-effectiveness

The total cost of care attributable to BSI before and after the intervention equals the sum of the mean patient and hospital costs per BSI neonate multiplied by the number of BSI patients in each group. The outcome yielded an estimated ICER of approximately $3,679 cost saving per neonatal BSI avoided (Table 6), suggesting that the intervention is a dominant strategy.

**Table 6:** Incremental cost-effectiveness.

	Admitted to ward with CHHC* (pre-intervention figures applied)	Admitted to ward with intervention (CHHC+OHHC**) (post-intervention figures applied)	Difference (Ratio of cost to effect)
**Parameter values**			
Neonate patients cohort	469	469	
Intervention costs	15,329	34,538^e^	
Probability of neonatal BSI	0.28	0.18	
Mean BSI-attributable patient costs	1,026	599	
Mean additional hospital costs	1,010	520	
**Results**			
Expected BSI-related costs			
Patient	134,734	50,536	
Hospital	132,633	43,898	
Total	267,367	94,434	172,933
Expected number of BSI-cases	131	84	47
ICER^β^			3,679

*Conventional hand hygiene care with soap and water;

**Optimal hand hygiene with alcohol-based hand rub

^e^Cost of OHHC, which equals 15,329 plus 19,209,

^β^ICER—Incremental cost-effectiveness ratio.

### Sensitivity analysis result

**Fig 2:**
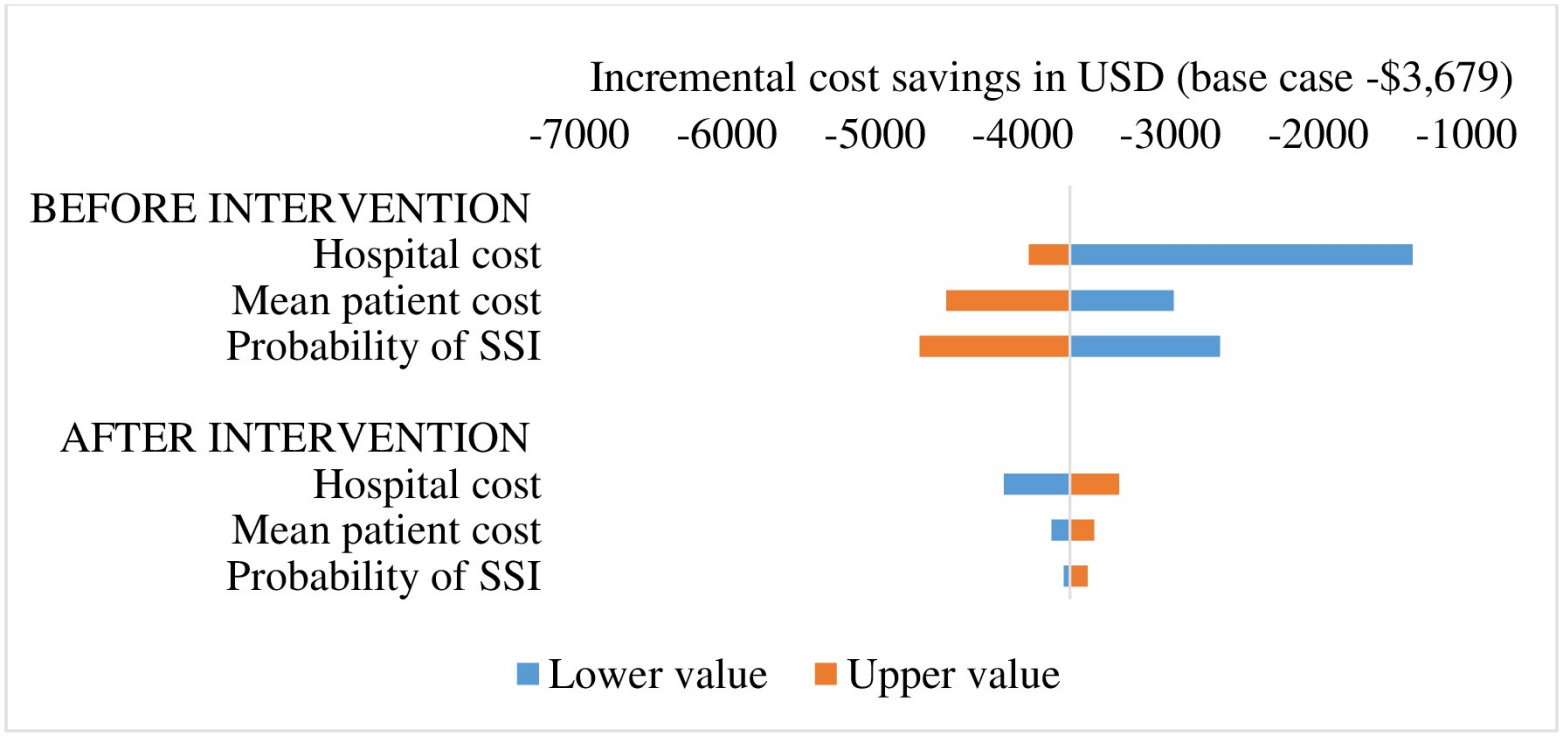
Incremental cost savings using upper and lower parameter values (base value -$3,679).

**Fig 3:**
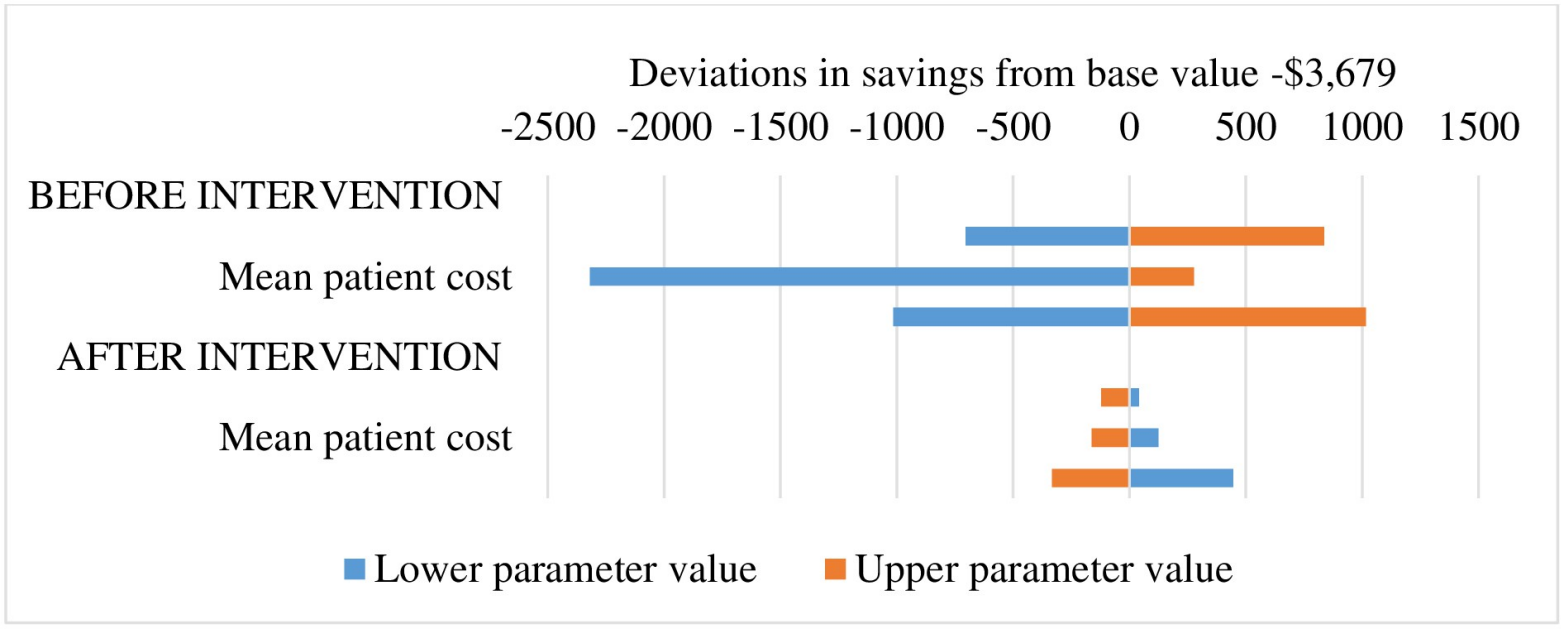
Deviations in savings from base value -$3,679.

Results from the sensitivity analysis shows the intervention may lead to cost savings if the input parameters are varied within the 95% uncertainty intervals. Fig 2 illustrates a possible maximum and minimum cost savings of -$4,695 and -$1,360 if the before-intervention probability of neonatal BSI and the hospital cost assume the maximum and minimum values of the 95% uncertainty intervals, respectively. Likewise Fig 3 also show that the maximum and minimum deviations from the incremental cost savings center around same parameters.

## Discussion

Before January 2018, the NICU provide only water and soap for hand hygiene. Water flow was infrequent, occasioned by rationing from the national grid and other technical challenges. Therefore, compliance with hand hygiene was becoming a challenge vis-à-vis alarming cases of hospital-acquired infections [4]. The WHO proposed multimodal hand hygiene involving alcohol-based hand rub for effective infection control [15]. Nonetheless, uptake of the recommended multimodal hand hygiene strategy in NICU settings is at extra cost to healthcare providers, a reason the NICU was unable to implement it before the HAI-Ghana study. In resource-limited settings like Ghana, decision-makers are interested in the most effective and cost-saving intervention when making an investment decision. Therefore, this study examines and compares the cost-effectiveness of a multimodal hand hygiene strategy involving alcohol-based hand rub plus soap and water versus only liquid antiseptic (soap and water) for infection control at an intensive care unit in a tertiary hospital in Ghana. The primary outcome measured was the number of avoided BSI episodes and the consequent cost savings from patients’ and provider perspectives.

The base case analysis of incremental outcomes suggests the intervention with ABH is a dominant hand hygiene strategy [23] that could significantly reduce neonatal BSI risk and further leads to a half reduction in extra LOS, among other indictors of BSI severity. Further, the quantified incremental patient cost savings indicate the ABH intervention has potential cost savings for patients, and healthcare providers. Deductively, society may save close to $196,000 in costs annually at the hospital alone. There is no published data on neonatal BSI incidence in Ghana as a whole, but applying the observed risk reduction to an estimated annual NICU admission of approximately 200,000 in Ghana, computed from recently published sources [24–26], will yield an annual patient cost savings of about $8.5 million. However, the associated cost savings may vary due to differences in the cost of living across the sixteen administrative regions in Ghana. For instance, transport cost is not uniform across the country, but it contributes 9.3% of the total cohort cost of hospitalization per BSI neonate at the Korle Bu Teaching Hospital.

We observed that the intervention is dominant and the incremental cost savings is, in descending order of magnitude, sensitive to some parameters, including the probability of BSI, hospital cost, and patient cost. Nonetheless, the incidence of neonatal BSI at the NICU may depend somehow on other circumstances such as the percentage formulation of the alcohol-hand rub, ring and artificial nail wearing by medical staff, among other environmental and individual factors [27, 28]. Also, including the most common antibiotics for treatment of neonatal BSI at the NICU i.e. Amikacin and Merrem/meropenem in the approved medicine list covered by the national health insurance scheme [29] could reduce situations where unstandardized pricing of those drugs contribute to the overall patient cost of neonatal BSI in Ghana, and consequently the burden of out-of-pocket payment cost.

Further, the observed fifty percent reduction in BSI-attributable extra LOS with the intervention is 6.5 days less than has been reported in other NICU settings [10]. However, the fifty percent reduction in LOS could result in an avoidable 1,953 patient bed days annually, and consequently, allow additional 171 admissions at the NICU. Likewise, we note that the intervention could result in 214 avoidable number of neonatal BSI at the NICU annually and 20,000 in total for Ghana. The potential gains could be more if the recommended protocol for multimodal hand hygiene strategy with alcohol based handrub was strictly followed above an observed rate of 60%. For instance, a review of studies on the impact of multimodal hand hygiene strategies show that strict adherence with alcohol-based handrub could reduce by half the incidence of nosocomial infections including neonatal BSI [10, 15].

Our case for investment consideration by policy-makers and healthcare providers is that assuming the intervention prevents 10% risk of neonatal BSI cases in Ghana as revealed in the base-case analysis, the estimated ICER suggests society can save in excess of $74 million annually.

## Strength and limitations of the study

### Strength

The strength of the study hinges on the quality and reliability of the data and the robustness of the methodology. The findings also provide a snapshot of reliable information on the trade-offs between two hand hygiene technologies for health policy decision-making, especially with regards to preventive interventions for nosocomial bloodstream infections among neonates in Ghana. To the best of our knowledge, this study is the first to do so in the context of Ghana using quality data from HAI-Ghana study.

### Limitations

The paper did not cover the generality of the health consequences associated with neonatal BSI in Ghana. The design and data limitation constrained our ability to estimate BSI-attributable disability-adjusted life years (DALYs) and quality-adjusted life-years (QALY) for our sample, which we anticipate may contribute to an underestimation of the cost and effect associated with the intervention. Again, we only compared the cost and outcomes for a short time and did not consider future costs. The use of average cost figures may not reflect the true impact of the intervention and patient spending. Our exclusion criteria meant we excluded four patients with severe congenital malformations and three patients who had undergone surgery, which could have nuanced the cost savings somehow. Also, the study was limited to one NICU setting due to funding constraints and may limit the ability to generalise the result to the entirety of Ghana. We anticipate that fundamental differences in the pricing of goods and services i.e. transport, accommodation, feeding, drugs, etc. may affect how much patient spend on their health. Therefore, such differences should be considered when looking at the macro impact of the intervention.

## Conclusion

The study examined the cost-effectiveness of alcohol-based handrub in preventing neonatal BSI in NICU settings. On the evidence of the analysis, the study shows positive gains with respect to cost savings and neonatal health outcomes. Though the cost of the intervention may be more relative to using just soap and water for hand hygiene, the outcomes is a good reason for policy-makers and healthcare providers to invest into the intervention to reduce the incidence of hospital-acquired neonatal BSI and the associated costs from patient and providers’ perspectives.

## Supporting information

S1 Data (XLSX)

## Abbreviations

ABH: Alcohol-based Handrub
BSI: Blood stream infection
CHEERS: Consolidated Health Economic Evaluation Reporting Standards
DALY: Disability-Adjusted Life-Years
HAI: Hospital-Acquired Infections
KBTH: Korle Bu Teaching Hospital
LMIC: Low and Middle-Income Countries
LOS: Length of Stay
NICU: Neonatal Intensive Care Units
QALY: Quality-Adjusted Life-Years
